# Novel *Bacteroides Vulgatus* strain protects against gluten-induced break of human celiac gut epithelial homeostasis: a pre-clinical proof-of-concept study

**DOI:** 10.1038/s41390-023-02960-0

**Published:** 2024-01-04

**Authors:** Tina Tran, Stefania Senger, Mariella Baldassarre, Rachel A. Brosnan, Fernanda Cristofori, Marco Crocco, Stefania De Santis, Luca Elli, Christina S. Faherty, Ruggero Francavilla, Isabella Goodchild-Michelman, Victoria A. Kenyon, Maureen M. Leonard, Rosiane S. Lima, Federica Malerba, Monica Montuori, Annalisa Morelli, Lorenzo Norsa, Tiziana Passaro, Pasqua Piemontese, James C. Reed, Naire Sansotta, Francesco Valitutti, Ali R. Zomorrodi, Alessio Fasano, CDGEMM Team, Maria Luisa Forchielli, Adelaide Serretiello, Corrado Vecchi, Gemma Castillejo de Villasante, Giorgia Venutolo, Basilio Malamisura, Angela Calvi, Maria Elena Lionetti, Mariella Baldassarre, Chiara Maria Trovato, Nicoletta Pietropaoli, Michela Perrone, Lidia Celeste Raguseo, Carlo Catassi

**Affiliations:** 1 Mucosal Immunology and Biology Research Center, Division of Pediatric Gastroenterology and Nutrition, Massachusetts General Hospital; Department of Pediatrics, Harvard Medical School, Boston, MA USA; 2 Center for Scientific Review, National Institutes of Health, Bethesda, MD USA; 3 NICU, University of Bari, Bari, Italy; 4 Pediatric Unit “Bruno Trambusti”, Osp Pediatrico Giovanni XXIII, University of Bari, Bari, Italy; 5 Department of Pediatrics, IRCCS Ospedale Giannina Gaslini, Genova, Italy; 6 Digestive Health Research Institute, Case Western Reserve University School of Medicine, Cleveland, OH USA; 7 Department of Pathology, Case Western University School of Medicine, Cleveland, OH USA; 8 Celiac Disease Referral Center, Ospedale Maggiore Policlinico, Milan, Italy; 9 Division of Pediatric Gastroenterology and Nutrition, Department of Pediatrics, Mass General for Children, Boston, MA USA; 10 Pediatric Gastroenterology Unit, Policlinico Umberto I, Sapienza University of Rome, Rome, Italy; 11 Pediatric Training Program, University of Salerno School of Medicine, Salerno, Italy; 12 Pediatric Hepatology Gastroenterology and Transplant Unit, Ospedale Papa Giovanni XXIII Bergamo, Bergamo, Italy; 13 Celiac Disease Referral Center, “San Giovanni di Dio e Ruggi d’Aragona” University Hospital, Pole of Cava de’ Tirreni, Salerno, Italy; 14 NICU, Fondazione IRCCS Ca’ Granda Ospedale Maggiore Policlinico, University of Milan, Milan, Italy; 15 Pediatric Gastroenterology and Liver Unit, Santobono-Pausilipon Children’s Hospital, Naples, Italy; 16 European Biomedical Research Institute of Salerno, Salerno, Italy; 17 Department of Medical and Surgical Sciences, University of Bologna, Bologna, Italy; 18 Unidad de Gastroenterología Pediátrica, Servicio de Pediatría, Hospital Universitari Sant Joan de Reus, Reus, Spain; 19 Celiac Disease Referral Center, “San Giovanni di Dio e Ruggi d’Aragona” University Hospital, Pole of Cava de’ Tirreni, Salerno, Italy; 20 Department of Pediatrics, Univ. Politec. delle Marche, Ancona, Italy; 21 Pediatric Unit “Bruno Trambusti,” Osp Pediatrico Giovanni XXIII, University of Bari, Bari, Italy; 22 Celiac Disease Referral Center, Bambino Gesù Hospital, Rome, Italy

## Abstract

**Background and aims:**

We have identified a decreased abundance of microbial species known to have a potential anti-inflammatory, protective effect in subjects that developed Celiac Disease (CeD) compared to those who did not. We aim to confirm the potential protective role of one of these species, namely *Bacteroides vulgatus*, and to mechanistically establish the effect of bacterial bioproducts on gluten-dependent changes on human gut epithelial functions.

**Methods:**

We identified, isolated, cultivated, and sequenced a unique novel strain (20220303-A2) of *B. vulgatus* found only in control subjects. Using a human gut organoid system developed from pre-celiac patients, we monitored epithelial phenotype and innate immune cytokines at baseline, after exposure to gliadin, or gliadin plus *B. vulgatus* cell free supernatant (CFS).

**Results:**

Following gliadin exposure, we observed increases in epithelial cell death, epithelial monolayer permeability, and secretion of pro-inflammatory cytokines. These effects were mitigated upon exposure to *B. vulgatus* 20220303-A2 CFS, which had matched phenotype gene product mutations. These protective effects were mediated by epigenetic reprogramming of the organoids treated with *B. vulgatus* CFS.

**Conclusions:**

We identified a unique strain of *B. vulgatus* that may exert a beneficial role by protecting CeD epithelium against a gluten-induced break of epithelial tolerance through miRNA reprogramming.

**Impact:**

- Gut dysbiosis precedes the onset of celiac disease in genetically at-risk infants.
- This dysbiosis is characterized by the loss of protective bacterial strains in those children who will go on to develop celiac disease.
- The paper reports the mechanism by which one of these protective strains, *B. vulgatus*, ameliorates the gluten-induced break of gut epithelial homeostasis by epigenetically re-programming the target intestinal epithelium involving pathways controlling permeability, immune response, and cell turnover.

## Introduction

In the past two decades, we have acquired a deep understanding of the adaptive immune response mechanisms involved in celiac disease (CeD) pathogenesis.^1–3^ However, there is still limited information regarding the initial steps causing the loss of gut mucosal tolerance to gluten and the switch from genetic predisposition to onset of the disease. Environmental factors^4,5^ have been shown to affect both the composition and function of the gut microbiome, and several reports have suggested a pathogenic role of specific microorganisms in CeD pathogenesis.^6–10^ However, evidence to mechanistically link either a single pathogen or gut dysbiosis to CeD pathogenesis is lacking. Moreover, the emerging role of epigenetic modulations such as microRNAs (miRNAs), has been identified in the pathophysiology of CeD,^11,12^ but just how these modulations are triggered remains to be elucidated.

To overcome these limitations, we initiated a prospective, longitudinal birth cohort study called the Celiac Disease Genomic, Environmental, Microbiome and Metabolomic (CDGEMM) study, which enrolls infants at-risk of CeD.^13^ We utilized this study to examine the infants’ microbiome and metabolome during the first 6 months after birth in relation to environmental risk factors and genetic markers known to increase susceptibility to CeD development.^14^ In a subsequent study, we investigated changes in microbiome and metabolomic profiles preceding the onset of CeD by up to 18 months.^15^ Among the differences identified, we detected the decreased abundance of several microbial species/strains known to have a potential anti-inflammatory, protective effect in cases (defined as at-risk children who developed CeD) before CeD onset compared to controls (defined as age-matched, at-risk infants who did not develop CeD).^15^ With the present study, we aimed to further investigate the potential protective role of some of these bacterial strains isolated from children at risk of CeD who were protected from the onset of the disease. Among the identified bacterial strains, we used a prototype *B. vulgatus* strain 20220303 A2 (referred to as *B. vulgatus*-A2*)* to perform epigenetic profiling and functional studies using human, patient-derived gut organoids to mechanistically establish a protective effect of bacterial bioproducts against gluten-dependent changes on human gut epithelial functions.

## Methods

### Bacterial isolation from gut microbiota

We used stool samples from two CDGEMM cases and two matched controls, obtained at disease onset and 6 months prior in cases and the corresponding time points in controls. The Prospector^®^, GALT Inc culturomic platform (isolationbio, San Carlos, CA) was used to isolate bacterial strains previously shown to be potentially protective against CeD onset.^15^ Further details, including demographics of the four patients included in bacterial culture and isolation are presented in the Supplementary Material and Method Section.

### Preparation of the bacterial cell free supernatant (CFS)

To optimize the growth conditions of strains of interest, we tested various bacterial culture media (see Supplemental data for more details). Given that *B. vulgatu*s-A2 grew the most efficiently in comparison to the other four strains tested, it was selected as a prototype for a more in-depth genomic analysis and for additional functional studies. We collected CFS at four optical densities (ODs), namely 1.0, 0.8, 0.4, and 0.1. Based on the OD_600_ readings, the bacteria grew most efficiently in Brain Heart Infusion (BHI) Broth. The subcultures were then centrifuged at 4000 rpm; supernatants were collected and filtered through a 0.4 mm syringe filter and then filtered again using a 0.2 mm syringe filter. The CFS obtained was then aliquoted and stored at −80 °C for future experiments (see analysis of CFS below). The bacterial pellets were flash frozen and used for genomic DNA sequencing.

### Bacterial sequencing and mutation analysis

Our isolated *B. vulgatus*-A2 strain underwent genomic DNA sequencing by the SeqCenter (Pittsburgh, PA). Basic variant analysis of paired-end reads was performed using breseq (version 0.36.1) to align and compare the genome to reference genomes as previously reported.^16,17^ Details on our mutation analysis are provided in the Supplementary Material and Methods Section.

### Human gut organoids and establishment and characterization of a macrophage-epithelium organotypic model

Experiments using organoid-derived monolayer from human gut and human monocytes were conducted as previously described^17^ after obtaining approval from the Massachusetts General Hospital Institutional Review Board protocol #2014P000198 (for additional details and patients’ selection for organoid preparation, see Supplementary data).

### CFS analysis design and procedures

After ~8 days in culture, organoid-derived monolayers reached confluency based on the reported Trans Epithelial Electrics Resistance (TEER) values as previously performed.^17^ The monolayers were then allowed to differentiate for 24 h. After differentiation, the media were changed to Dulbecco’s Modified Eagle Medium (MilliporeSigma, Darmstadt, Germany) (DMEM)/F12 (without FBS) both apically and basolaterally, and 10% CFS of the *B. vulgatus*-A2, 10% heat-inactivated CFS, or 10% BHI Broth (control) were added apically to the monolayers and then incubated at 37 °C for 24 h. Half of the CFS- and BHI-treated monolayers received apically 1 mg/ml of a peptic-tryptic digest of gliadin (PTG) 4 h into the incubation. Following the CFS and PTG exposure time, cellular permeability and cytotoxicity were measured, and the basolateral supernatants of the monolayers were collected for cytokine secretion analyses. The experiments were repeated six times.

### Measurement of monolayer permeability

TEER was measured daily to monitor the organoid-derived monolayer development as previously reported [18]. Paracellular permeability was also measured based on fluorescein isothiocyanate-polyethylene glycol (FITC-PEG) 550 Da (#PG1-FC-550, Nanocs, Boston, MA) diffusion across the monolayers.^17^ As previously shown, fluorescence absorbance is directly proportional to the amount of FITC-PEG that crossed the monolayers and correlates directly with the paracellular permeability of the monolayers.^18^

### Measurement of cellular viability

A lactase dehydrogenase (LDH) assay, Cytotoxcity Detection Kit^PLUS^, #0474493001 (MilliporeSigma) was used according to the manufacturer’s protocol to evaluate the viability of the cell monolayers by testing the basolateral media from the transwell system. Cytotoxicity data are reported as a percentage of the absorbance of the untreated control. The background was subtracted from all the LDH release data. The percent cytotoxicity was calculated by dividing the experimental LDH release by the average of the untreated control with values greater than 100% interpreted as a reduction in cell viability.

### Baseline gene expression in organoids

Comparative gene expression analysis of healthy control (HC), acute CeD (ACD), celiac remission on a gluten-free diet (GFD), and potential CeD (PceD) were performed on organoids at baseline after 5 days in culture. RNA extraction was performed according to the manufacturer’s protocol. The RNA was purified with a Direct-zol RNA Miniprep Kit (#R2052, Zymo Research, Irvine, CA) per manufacturer’s instructions. cDNA was then synthesized from the purified RNA using the PrimeScript™ RT Master Mix (#RR036A, Takara Bio, San Jose, CA). The oligonucleotide primers employed for the RT-PCR analysis (Supplementary Table ST1) were designed by the MGH primer bank (Boston, MA) and synthesized by Integrative Device Technology (IDT, San Jose, CA). PerfeCTa SYBR Green SuperMix #95054-02 K (Quantabio, Beverly, MA), was used for the qPCR reactions. The quantitative RT-PCR was performed using a QuantStudio™ 3 Real-Time PCR System (Life Technologies, Carlsbad, CA). The 18S gene was used as an internal control for the experiments. The results were analyzed using the CT method (2^-∆∆CT^) and reported as a fold change value against the experimental control.

### Cytokine assay

Innate immune response-related cytokines were measured in the basolateral supernatants of the human gut organoid-derived monolayers and were measured using the Human Innate Immune panel chips (Isoplexis, Inc product code CODEPLEX-2L03-2), which were loaded into the IsoSpark system as previously described.^19^ The panel of secreted cytokines was analyzed using the IsoSpeak software 2.9.0. The amount of secreted cytokines for each treatment is reported as picograms per milliliter (pg/ml).

### Organoid monolayer microRNAs analysis

Total RNA from HC and PceD gut monolayers with or without *B. vulgatus*-A2 CFS treatment was extracted as previously reported^17^ and microRNA analysis performed as outlined in the supplementary data.

### Statistical analysis

All statistical analyses were performed with GraphPad Prism 9.0. Data set outliers were identified with the Robust regression and Outlier removal (ROUT) Method with a Q value of 1%. Data were analyzed with the ordinary one-way ANOVA. The Dunnett’s multiple comparisons test with a single pool variance was used to identify significance levels within the data points. For the organoid monolayer microRNAs analysis, data were expressed as means ± standard error of the mean (SEM) and statistical significance were evaluated with two-tailed t-test. Significance levels were recognized by *p* < 0.001, *p* < 0.01, and *p* < 0.05.

## Results

### Bacterial isolation

Evidence suggests that the biological effect of commensal organisms may be associated with differences detected at the strain level.^20^ Thus, we decided to investigate the effect of specific strains isolated from stool samples of the CDGEMM subjects, rather than relying on publicly available strains. Using a high throughput, microbial cultivation system (Prospector^®^, GALT Inc.), we isolated 3500 bacterial colonies from stool samples from two CeD cases and two matched controls at the time of the onset of CeD and 6 months prior, resulting in approximately 150 unique strains. We identified five strains that we had previously found to be specifically enriched in control fecal samples compared to CeD cases,^16^ which were subcultured for further investigation. The selected strains included *Bifidobacterium breve*, shown to have significant immune regulatory activity;^21^ *Bacteroides thetaiotaomicron*, reported to be protective in a preclinical model of IBD;^22^ *Bifidobacterium longum*, recognized to have protective functions on cell barrier and cytokine modulation;^23,24^ *Bacteroides vulgatu*s, reported to ameliorate intestinal inflammatory diseases in a DSS-induced model of colitis;^25^ and *Bacteroides uniformis*, reported to improve immune defense mechanisms in a preclinical model of obesity.^26^

### Genotypic, molecular, and phenotypical characterization of pre-onset human gut organoid

**Fig. 1:**
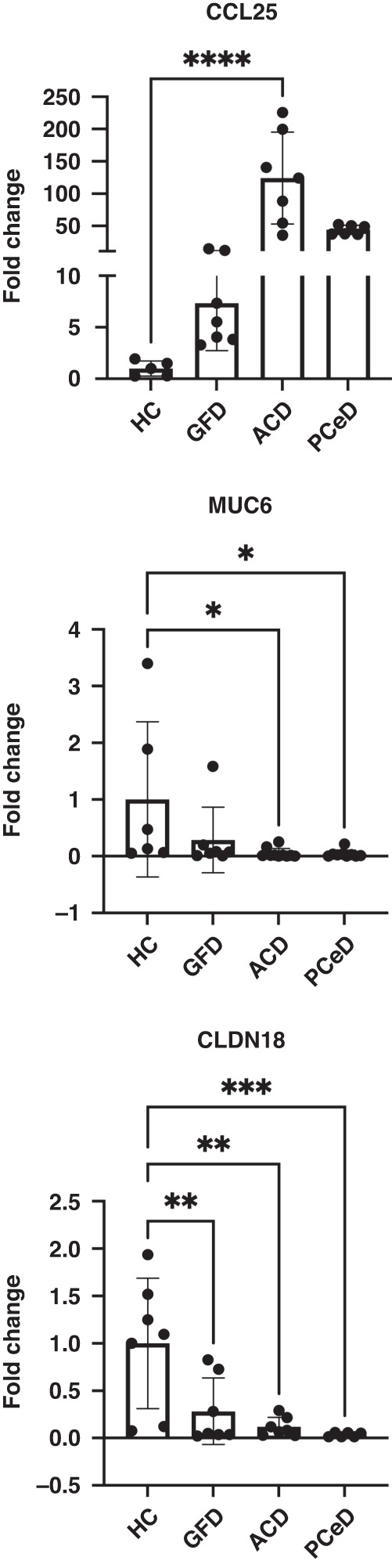
Fold change (FC) of signature genes expressed in organoids of potential CeD (PCeD) compared to healthy controls (HC, *N* = 2), acute (ACD, *N* = 2) and remission (GFD, *N* = 2)). Markers of acute celiac activation related to goblet cells signature, chemokines and barrier [SDS1] were found already altered in both PCeDs. Triplicate for each organoid. One-way ANOVA. (*P* values: *<0.05, **<0.01, ***<0.001), ****<0.0001.

Gene expression comparative analyses (Fig. 1) revealed that the prototype *PceD* organoids tested (PceD1) had altered gene expression levels for a subset of markers associated with goblet cells, tight junctions and chemokine signaling cascades consistent with our previous observations in acute celiac organoids (ACD).^18^ Similar alterations observed in PceD1 were confirmed in an organoid derived from a second pediatric PceD (PceD2) patient not enrolled through the CDGEMM study (Fig. 1). Other analyzed genes, including FUT-1, MUC2, and IL37 showed no differences (data not shown). These data suggest that, despite the absence of enteropathy, the duodenal epithelium from PceD patients had already shifted, at least partially, toward an altered gene expression profile typical of the acute phase of CeD months before its onset. Alternatively, subjects at risk of developing CeD may have a specific genetic predisposition (direct or indirect) that influences the basal expression of the aforementioned genes in PceD.

### Effect of microbial CFS on patients’ gut epithelium-MΦ co-culture in response to gliadin exposure

**Fig. 2:**
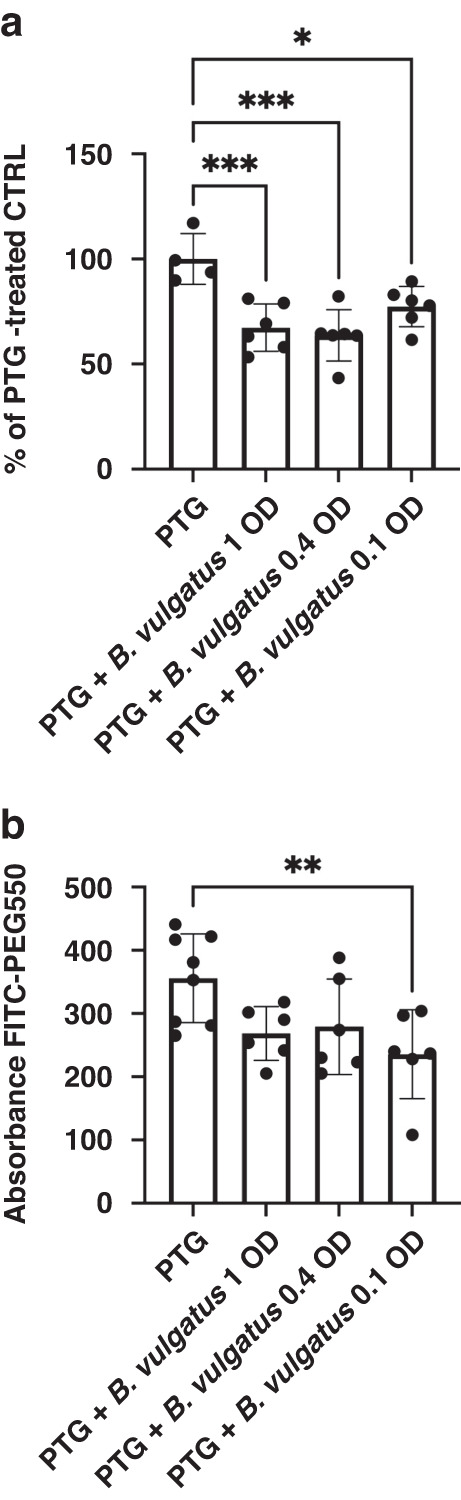
The protective effect of *B. Vulgatus*-A2 CFS against PTG-induced cell death and increased epithelial paracellular permeability is dose-dependent. Dose-dependent effect of *B. vulgatus-A2* CFS on cell viability (**a**) and on epithelial paracellular permeability (**b**) of PCeD organoids (*N* = 2) exposed to PTG. *N* of experiments/organoid = 3. One-way ANOVA test (*) *P* < 0.05, (***)*P* < 0.001, (****) *P* < 0.0001. (**) *P* < 0.01, (***) *P* < 0.001.

Gut mucosal response to environmental factors, including gliadin, is the result of a coordinated epithelial-immune action. Following the validation of our organoid-derived monolayer from human gut-MΦ co-culture (see supplemental data and Supplementary Fig. S1), we evaluated CFS derived from five candidate strains (see above) isolated from the CDGEMM control subjects to explore potential modulatory effects on the PceD GI epithelium-MΦ response when exposed to gliadin. When cell monolayers were treated with the five tested isolates CFS, *B. longum*, *B thetaiotaomicron* and *B. vulgatus* were the most consistent in mitigating the monolayer permeability, whether induced by gliadin (Supplementary Fig. S2) or at baseline (not shown) in PceD organoid-derived monolayer from human gut. Of note, *B. vulgatus* CFS was also able to improve the epithelial cell viability (Fig. 2a) in addition to ameliorating increases in gliadin-induced permeability (Fig. 2b). Protective activity of the *B. vulgatus* CFS correlated directly with the density (log phase) of the culture of origin (Fig. 2). Finally, when we compared the effect of microbial CFSs on organoids’ monolayers versus epithelium-macrophage organotypic cultures (data not shown), we did not observe differences in paracellular permeability and cytotoxicity readouts, suggesting that the observed protective effect was not mediated by macrophages. Combined, these observations prompted us to further investigate *B. vulgatus*-A2 CFS activity on the epithelium.

### *B. vulgatus* sequencing and genomic analysis

We assessed whether the *B. vulgatus*-A2 strain was the same strain available in reference databases or contained unique features. To this end, we sequenced the genome of our isolated *B. vulgatus*-A2 strain and compared its assembled genome with the genome of *B. vulgatus* ATCC 8482 reference strain. A list of over 20,000 mutations was identified in the newly isolated strain compared to *B. vulgatus* ATCC 8482. In particular, two large deletions were noted in this strain, a 78-gene segment starting at base pair 4,200,749 and an 85-gene segment starting at base pair 920,657. Notably, the latter included a gene for lipopolysaccharide (LPS) biosynthesis related polysaccharide transport and flippase (A6KY90).

The 20,000 mutations list was reviewed, and mutations in areas that were not of interest were removed from further investigation (see Supplementary Methods for details). A complete list of annotations for these removed genes is reported in Supplementary Fig. S3. This process yielded 1369 remaining mutations in 1342 distinct genes. These genes were involved in several functions in bacteria according to UniProt annotations (see Supplementary Methods and Supplementary Fig. S4). We further investigated these genes for either direct or indirect interactions of the associated gene products with host cells, such as modulating gut barrier function, or immune response and inflammation. Specifically, we found that several genes responsible for the secretion of bacterial proteins and metabolites contained transglutaminase domains (a key enzyme in CeD pathogenesis), or were involved in quorum sensing based on gene annotations or literature review. Furthermore, we conducted an automated search in PubMed (see Methods) to mine the literature for any evidence that these 1342 genes might be implicated in interactions with the human host. The identified articles from this automated search were then reviewed and the findings were combined with the results from the manual inspection of the mutations. The combined approaches identified a total of 39 genes involved in interactions with the host according to gene annotations and/or prior literature (Table 1). Out of these 39 genes, 13 had products that were secreted or exported, which could alter the microbe–host shared environment. Some examples include an AsmA-like protein involved in glycerophospholipid transport and bacterial outer membrane integrity (A6KWC8), the RND subunit of a putative metal resistance exported protein (A6L1Y6), and a sulfatase/hydrolase that can stimulate pro-inflammatory responses in the host (A6KZU2).^27–29^ Another notable example of a mutated gene encoding an exported protein in our isolated strain was a gene encoding a protein with transglutaminase activity that could be involved in regulating bacterial cell death and host cell apoptosis (A6KWH9).^30^ We additionally identified a second transglutaminase domain-containing protein involved in production of antibody and drug conjugates via transpeptidation (A6L738).^31^ Although mutations in these genes with transglutaminase activity were observed in *B. vulgatus*-A2, the proteins could impair the epithelial transglutaminase-driven deamidation reaction of gliadin that is presented by the MHC HLA DQ2/8 complexes to CD4^+^ T cells, a key step leading to the typical CeD autoimmune enteropathy. Examples of additional mutations are listed in the supplemental material. It should be noted that the protective change observed in our experiments for the isolated *B. vulgatus*-A2 strain might be due to the effect of mutations in a combination of these 39 genes and not necessarily due to mutations in individual genes.

**Table 1:** The composite list of 39 genes from the automated PubMed search and manual review of the 1342 gene mutations.

Uniprot ID	Base Pair	Identification Method	Annotation	Grouping	Citations
Gene Products with Direct Effects					
A6KWC8	5975	Manual/Automated	Putative exported protein, AsmA-like - these are critical for glycerophospholipid transport and outer membrane integrity	Barrier Function	35226662
A6KWH9	91,425	Manual	Conserved hypothetical exported protein with transglutaminase activity that could be involved in regulating cell death and apoptosis	Inflammation	24464646
A6KWJ8	117,182	Manual	Putative secreted protein involved in glyocsidic bond hydrolysis	Unknown	8535779
A6KX36	412,558	Manual	Putative exported protein	Unknown	
A6KZ75	1,395,966	Manual	Putative secreted sulfatase	Inflammation, Metabolism and Nutrient Processing	186484, 3982438
A6KZD5	1,476,083	Manual	Putative exported D-alanyl-D-alanine carboxypeptidase penicillin-binding protein (DacB)	Metabolism and Nutrient Processing	32625007
A6KZU2	1,700,527	Manual	Putative exported sulfatase and hydrolase that can stimulate pro-inflammatory response in host	Barrier Function, Inflammation	25974305
A6L0C6	1,930,429	Manual	Secreted protein involved in carbohydrate and protein metabolism, BACON domain containing	Metabolism and Nutrient Processing	20416301
A6L0D9	1,945,454	Manual	Putative secreted tripeptidyl aminopeptidase which cleaves terminal amino acids	Metabolism and Nutrient Processing	8440407
A6L198	2,298,035	Manual	Secreted protein	Unknown	
A6L1Y6	2,636,133	Manual	Putative metal resistance related exported protein, RND Subunit	Barrier Function	27806930
A6L240	2,706,777	Manual	Putative exported cytochrome C biogenesis-related protein	Barrier Function, Metabolism and Nutrient Processing	11967064, 12524212
A6L2T7	3,018,133	Automated	Peptidoglycan hydrolase (LysM)	Barrier Function, Cell Replication and Turnover, Inflammation, Metabolism and Nutrient Function	27708039
Gene Products with Indirect Effects					
A6KWC3	74,433	Automated	NigD‚-like protein, a lipoprotein	Unknown	19531060, 35871068
A6KWI0	92,576	Manual	Signal peptidase I, a protein that cleaves hydrophobic, N-terminal signal or leader sequences from secreted and periplasmic proteins allowing for release from the membrane	Inflammation	22031009
A6KWM3	153,245	Automated	Putative outer membrane protein from the TonB receptor family probably involved in nutrient binding and shown to polarize host macrophages into the M2 state (DnaK)	Barrier Function	25419575
A6KXR1	732,950	Manual	Neuraminidase, a BNR repeatcontaining protein and possible virulence factor	Inflammation, Metabolism and Nutrient Processing	7463468
A6KXX6	831,953	Automated	4-O-beta-D-mannosyl-D-glucose phosphorylase, converts 4-O-beta-D-mannopyranosyl-D-glucopyranose (Man-Glc) to mannose 1-phosphate (Man1P) and glucose	Barrier Function, Metabolism and Nutrient Processing	23954514
A6KYC8	1,014,004	Automated	Alpha-galactosidase, Hydrolysis of terminal, non-reducing alpha-D-galactose residues in alpha-D-galactosides, including galactose oligosaccharides, galactomannans and galactolipids	Metabolism and Nutrient Processing	36090029
A6KYS6	1,202,941	Manual	S-ribosylhomocysteine lyase, involved in the synthesis of secreted autoinducer 2 (AI-2) used in quorum sensing, biofilm formation, and virulence factor expression	Cell Replication and Turnover, Inflammation	24026770
A6KZ59	1,363,629	Manual	Putative transporter, methyltransferase domain containing	Barrier Function	
A6KZ98	1,423,337	Automated	Glycosyltransferase	Metabolism and Nutrient Processing	
A6KZV5	1,722,822	Automated	Clostripain-related cysteine peptidase	Metabolism and Nutrient Processing	
A6L0P3	2,060,343	Automated	Conserved protein (TraN) found in conjugation transposon involved in antagonistic secretion systems to compete with other *Bacteroides* species	Cell Replication and Turnover	26768901
A6L100	2,173,954	Automated	GTPase Obg (ObgE), an essential GTPase which binds GTP, GDP and possibly (p)ppGpp. Plays a role in control of the cell cycle, stress response, ribosome biogenesis and in those bacteria that undergo differentiation, in morphogenesis control	Cell Replication and Turnover, Metabolism and Nutrient Processing	34284824
A6L2L7	2,939,217	Automated	Amidohydrolase	Metabolism and Nutrient Processing	
A6L3C3	3,234,698	Automated	Phosphotransferase, APH domain containing protein	Metabolism and Nutrient Processing	
A6L3P3	3,396,727	Manual	Putative exopolymeric substances related membrane protein, involved in physiologic stress response	Barrier Function	
A6L3S7	3,416,804	Automated	Transporter	Barrier Function	
A6L4A1	3,638,999	Automated	Putative outer membrane protein, tryptophanrich sensory protein	Barrier Function	32937127
A6L4B2	3,650,452	Automated	Ferrous iron transport protein B that participates in iron chelation, reducing inflammation in UC patients	Barrier Function	27724868, 12379679
A6L4G6	3,736,170	Automated	Transcriptional regulator	Cell Replication and Turnover	
A6L4I5	3,756,653	Automated	DNAbinding protein, HU-HIG domain containing protein	Cell Replication and Turnover	
A6L4R8	3,853,411	Automated	LysM peptidoglycanbinding domaincontaining protein	Unknown	27708039
A6L4U7	3,889,712	Manual	Putative exported periplasmic protein, ATP binding cassette transporter	Metabolism and Nutrient Processing	26517916
A6L4W1	3,908,142	Manual	Putative exported periplasmic protein, ATP binding cassette transporter	Metabolism and Nutrient Processing	26517916
A6L738	4,807,737	Manual	Transglutaminase domaincontaining protein involved in production of antibody and drug conjugates via transpeptidation	Metabolism and Nutrient Processing	31643050
A6L7K9	5,005,462	Manual	Tricorn protease homolog	Metabolism and Nutrient Processing	11347893
A6L7U1	5,117,642	Automated	ABC transporter ATP‚-binding protein/permease	Barrier Function	19302324

The table is divided into gene products that could exert a protective phenotype either directly, e.g., an exported protein, or indirectly, e.g., a transcriptional activator affecting the level of a gene product being produced. Two large deletions of 78 and 85 genes described in the results section were not included in this table.

### Effect of *B. vulgatus-A2* CFS on gliadin-induced cytokine release in human gut epithelium

**Fig. 3:**
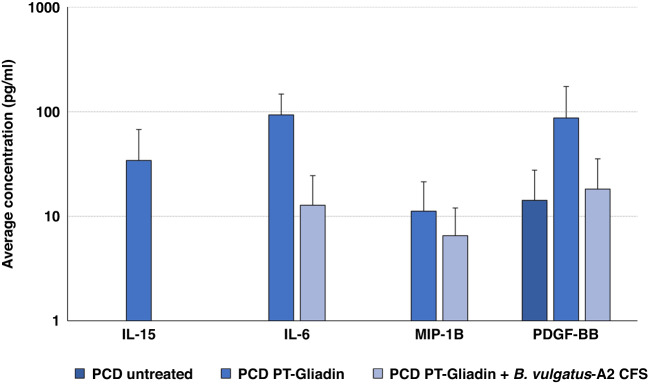
Cytokine release analysis (*N* = 2 per treatment) with average bars in pg/ml from two bio replicates, each with technical duplicates. Error bars are representative of the standard deviation of the averages.

To establish the impact of *B. vulgatus*-A2 CFS on the response of the PceD gut epithelium, we measured innate immunity cytokines at baseline (before PT-gliadin exposure), after PT-gliadin exposure, and after PT-gliadin plus *B. vulgatus*-A2 CFS exposure. At baseline, several cytokines were constitutively secreted, including EGF, GM-CSF, IL4, PDGF-BB, sCD137, TNFa, and VEGF (Fig. 3 and Table 2). Following PT-gliadin exposure, there were increases in secretion of several cytokines, including IL-15, IL-6, MIP-1b, and PDGF-BB, which were completely prevented (IL-15) or reduced (IL-6, PDGF-BB, MIP-1b) by the exposure to *B. vulgatus*-A2 CFS (Fig. 3). Interestingly, *B. vulgatus*-A2 CFS was also able to completely ablate the constitutive TNFɑ secretion (Table 2). No effect was detected on the remaining cytokines analyzed (Table 2).

**Table 2:** Cytokine profiling at baseline, after PT-gliadin exposure and PT-gliadin plus *B. vulgatus*-A2 CFS in PCeD organoids.

Experimental Condition	EGF	GM-CSF	IL-10	IL-4	IL-8	IP-10	MCP-1	MIP-1a	sCD137	TNF-a	VEGF
PCD Untreated	47.91	35.09	2.27	15.60	2.40	7.73	ND	ND	53.43	23.35	171.11
PCD PT-Gliadin	135.9	15.37	13.39	13.96	71.13	15.95	5.74	ND	80.67	8.42	576.40
PCD PT-Gliadin + *B. vulgatus*-A2 CFS	92.11	7.62	15.15	13.06	76.18	29.94	10.71	18.24	63.49	ND	419.93

Analysis was performed by isoplex method and expressed in pg/ml of cytokine present in the basolateral organoid media.

### Effect of *B. vulgatus-A2* CFS on human gut organoid monolayer miRNAs

**Fig. 4:**
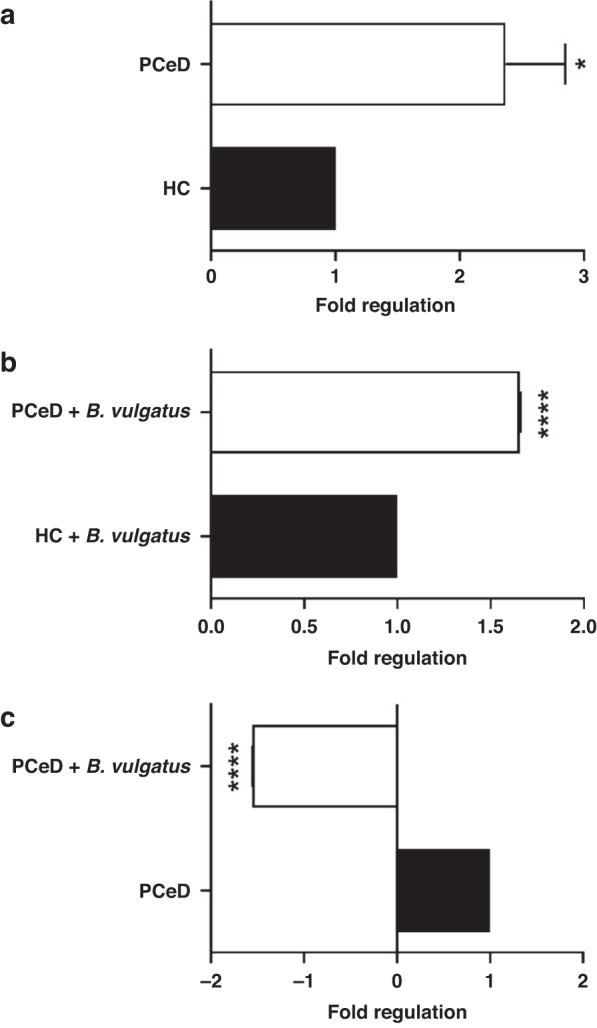
Effect of *B. vulgatus*-A2 CFS on miR-152-3p expression in both PCeD and HC organoids. High-throughput miRNAs analysis was performed by Real-Time PCR using pre-custom plates for the inflammatory response and the autoimmunity focus in PCeD vs. HC organoids at basal condition (**a**) and after *B. vulgatus*-A2 CFS treatment (**b**), and in PCeD organoids treated with *B. vulgatus*-A2 CFS vs. untreated ones (**c**) (*n* = 3 organoids derived monolayers/group). Histograms represent the mean ± SEM. *P < 0.05, ****P < 0.0001.

**Fig. 5:**
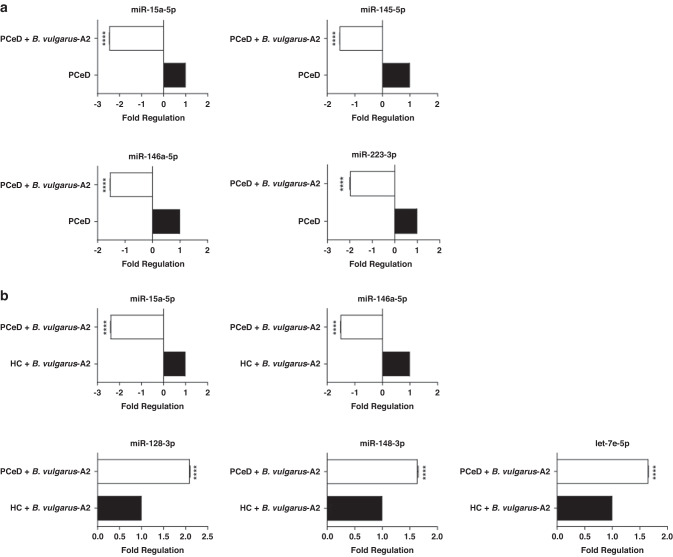
Effect of *B. vulgatus*-A2 CFS on miRNAs expression in both PCeD and HC organoids. Modulation of miRNAs expression in *B. vulgatus*-A2 CFS-treated PCeD organoids (white bars) vs. untreated PCeD organoids (black bars) (**a**). Differential expression of miRNAs in HC (black bars) and PCeD organoids (white bars) after the treatment with *B. vulgatus*-A2 CFS (**b**). For both the analyses, a high-throughput miRNAs analysis was performed by Real-Time PCR using pre-custom plates for the inflammatory response and the autoimmunity focus (n = 3 organoids derived monolayers/group). Histograms represent the mean ± SEM. ****P < 0.0001.

To complement the strain genome and functional analyses, we performed a high-throughput miRNAs analysis in organoid monolayers cultured from HC and PceD patients with or without *B. vulgatus*-A2 CFS treatment. Our results showed that miRNA modulations are mainly induced by this treatment; in fact, at basal condition the only significantly modulated miRNA is miR-152-3p whose expression is constitutively higher in PceD organoid monolayers when compared to HC organoid monolayers (Fig. 4a). Interestingly, the same miRNA seems to be specifically modulated by *B. vulgatus*-A2 CFS treatment. When the treatment with *B. vulgatus*-A2 CFS was performed, the expression of miR-152-3p showed less induction in PceD vs. HC organoid monolayers (Fig. 4b). Focusing on PceD organoid monolayers, an inverse trend for miR-152-3p modulation was reported after *B. vulgatus*-A2 CFS treatment (Fig. 4c). Furthermore, the *B. vulgatus* CFS treatment decreased the expression of miR-15a-5p, miR-145-5p, miR-146a-5p, and miR-223-3p in intestinal organoid monolayers from PceD (Fig. 5a). The same inhibitory effect on miRNAs expression was also reported for miR-146a-5p, and miR-15a-5p when organoid-derived monolayers from PceD were compared to those from HC, both exposed to *B. vulgatus* CFS treatment (Fig. 5b). The comparison between *B. vulgatus*-A2 CFS-treated organoids from PceD vs. *B. vulgatus*-A2 CFS-treated HC organoid monolayers also showed the induced expression of miR-128-3p, miR-148-3p, and let-7e-5p (Fig. 5b). On the contrary, another member of the let-7 family (let-7c-5p) was repressed in HC organoid monolayers after *B. vulgatus*-A2 CFS treatment (Supplementary Fig. S5). Of note, none of these miRNAs were modulated at baseline, highlighting the role of *B. vulgatus*-A2 CFS in regulating miRNAs expression in organoid monolayers. Based on target prediction and gene set enrichment analyses, all the miRNAs significantly modulated expression correlate with the involvement of signaling pathways regulating pluripotency of stem cells, MAPK, mTOR, and TGFβ signaling pathways (Supplementary Fig. S6). In addition, both these analyses showed the modulation of other inflammatory pathways for miR-146a-5p, such as the NF-κB and the T cell receptor signaling pathway (Supplementary Fig. S6).

## Discussion

While gluten serves as the main environmental trigger for CeD onset, we now appreciate that additional stimuli are necessary to lose tolerance to gluten and start the transition from genetic predisposition to clinical presentation. Among other environmental elements, special attention has been paid over the years to gastrointestinal infections. One of the first reports from almost 40 years ago hypothesized adenovirus infection as a possible trigger of CeD onset based on a possible antigen mimicry mechanism.^32^ Since then, several reports have been published claiming a pathogenic role of specific microorganisms in CeD onset.^10^ While this pathogenic mechanism remains a possibility, a likely alternative mechanism could involve a more complex disruption of the ecosystem of the gastrointestinal microbiota caused by infection rather than by the specific effect of a single pathogen on gut mucosal homeostasis. To explore this hypothesis, we developed a human pre-onset gut organoid model as a proof-of-concept approach to establish the epithelial response to gliadin exposure and to examine the potential protective role of bacterial strains found enriched in infants at risk of CeD who were protected against the onset of the disease compared to at-risk infants who lost tolerance to gluten and developed the disease. Among the five strains identified, we focused our studies mainly on *B. vulgatus*-A2, which showed sustainable growth and biological activity across all tested cultures in affecting the effect of gluten on gut epithelial homeostasis occurring during pre-clinical onset of CeD. Our data suggest that *B. vulgatus*-A2 strain may exert a protective role by acting on the gut epithelium with a coordinated action involving protection against loss of barrier function, cell death and pro-inflammatory cytokine production. These effects seem to correlate with an epigenetic reprogramming of the epithelial cells affecting these protective functions. Also interesting is the mutation of *B. vulgatus*-A2 transglutaminase activity, a feature that deserves further characterization, given the hypothesized role of bacterial-derived transglutaminases in CeD pathogenesis.^33^

At the molecular level, we have previously shown that gluten-derived peptides signal through MYD88-dependent activation of the CXCR3 receptor^23^ to promote secretion of IL-8^34^ and upregulation of paracellular permeability in the epithelium via zonulin release.^35^ We also generated data suggesting the involvement of MΦ in the early steps of CeD pathogenesis.^34,36^ Thus, we have adopted an in vitro organotypic, co-culture model that incorporated both epithelial cells and MΦ to establish whether specific epithelium-MΦ interactions are necessary to maintain homeostasis in the context of a microbiome-established micromilieu. Our data suggest that mucosal homeostasis does not require this cooperation and is maintained by the action of specific microbiota-secreted components that exert the double action of epigenetically influencing key functions of the host epithelium involved in antigen trafficking, cell turnover, inflammatory immune response, and protecting against gliadin-induced cell death and increased permeability through secreted molecules present in conditioned media. Using *B. vulgatus*-A2 strain as a prototype, we have shown that this unique strain isolated from controls and not present in cases had several mutations compared to the ATCC 8482 reference strain that affects its capacity to regulate metabolic pathways of the host related to inflammatory response and barrier function. These data were complemented by the phenotypic observation in the gut epithelium in which we detected that *B. vulgatus*-A2 CFS was able to ameliorate the gliadin-induced cell damage and increased epithelial permeability.

Moreover, the epigenetic changes align with the phenotypic outcome. Specifically, we have shown that organoid-derived monolayers from PCeD downregulated the expression of miR-15a-5p, miR-145-5p, miR146a-5p, and miR-223-3p after treatment with *B. vulgatus*-A2 CFS. These data are in accordance with the previous literature,^37–39^ except for miR-145-5p for which a reduced expression was reported in CeD patients.^40^ Target prediction and gene set enrichment analyses linked the four miRNAs to signaling pathways, which have been previously shown to be involved in CeD pathogenesis.^41–44^ Specifically, in line with the literature, the reduction of miR-15a-5p and miR146a-5p after *B. vulgatus*-A2 CFS treatment in PCeD when compared to HC organoid monolayers impairs TGF-β-mediated signaling, thus promoting and sustaining intestinal inflammation in CeD.^45^ Furthermore, the downregulation of miR-15a-5p induced by *B. vulgatus*-A2 CFS treatment correlates with the bacterial ability to ameliorate the gliadin-induced increased mucosal permeability in gut organoid monolayers. In fact, miR-15a-5p negatively regulates intestinal epithelial tight junctions through cell division cycle (Cdc)42 in pediatric IBD.^46^ Additionally, the treatment with *B. vulgatus*-A2 CFS increased the expression of miR-128-3p, miR148-3p, and let-7e-5p in PCeD organoids relative to HC organoid monolayers thus supporting a possible role for these miRNAs in promoting CeD pathogenesis. In accordance with the let-7e-5p data, another member of the let-7 family (let-7c-5p), which is known to regulate proliferation, differentiation and apoptosis in development and cancer,^47^ was reduced in *B. vulgatus*-A2 CFS-treated HC organoids compared to untreated HC organoids. The effect on let-7 family members seems to be mediated by *B. vulgatus*-A2 CFS; in fact, a significant decrease of circulating let-7e-5p was reported between controls and samples taken <1 year before tissue transglutaminase antibodies positivity.^48^ In addition, our data demonstrated the ability of *B. vulgatus*-A2 CFS treatment to specifically affect miR-152-3p expression as indicated by less upregulation of this miRNA in PCeD organoids compared to HC organoids before and after *B. vulgatus*-A2 CFS treatment. This trend was confirmed by the reduced expression of miR-152-3p in *B. vulgatus*-A2 CFS-treated organoids from PCeD. Even if miR-152-3p has a positive effect on CeD signaling pathways in different pathological contexts,^49,50^ to the best of our knowledge, no studies have been reported in literature that specifically correlate this miRNA to CeD. Interestingly, in our dataset miR-152-3p is the only miRNA to be significantly modulated in PCeD versus HC organoids at basal condition.

Target prediction and gene set enrichment analyses linked the four miRNAs to signaling pathways regulating the pluripotency of stem cells, MAPK, mTOR, and TGFβ, which have been previously shown to be involved in CeD pathogenesis.^41–48^ These signaling pathways involving immune response and regulation of barrier function are complementary with our findings on the *B. vulgatus*-A2 strain genetic mutations and our functional studies that we have reported above, further supporting a possible probiotic profile of this strain. Thus, our data suggest that the *B. vulgatus*-A2 strain protective effect against gluten shown by the phenotypic experiments could act through miRNAs reprogramming in the celiac gut epithelium. However, more studies are needed to confirm and integrate the miRNAs data reported in this study and to validate the predicted targets.

In conclusion, our in vitro modeling of the pre-onset phase of CeD suggests a break of gut epithelial homeostasis secondary to gut dysbiosis characterized by loss of protective bacterial elements that both epigenetically and functionally influence the gut epithelium response aimed at maintaining a state of energy toward gluten. Perturbation of this microbiota balance, secondary to unknown stressors, may lead to a break of tolerance to gluten, starting the transition from genetic predisposition to active disease. Our data provide preliminary potential evidence of possible targets for disease interception in subjects at risk of CeD who present with elements of dysbiosis in their pre-clinical phase. However, to translate our proof-of-concept evidence into implementable therapeutic strategies, additional corroboration of our results also exploring the combined cooperation between protective vs. offensive elements that characterize the microbiome “signature” of children at-risk that went on to develop CeD is needed.

## Disclaimer

This article was prepared while Stefania Senger was employed at Massachusetts General Hospital. The opinions expressed in this article are the author’s own and do not reflect the view of the National Institutes of Health, the Department of Health and Human Services, or the United States government.

### Supplementary information

Supplemental Data

## Data Availability

Materials described here and all relevant raw data will be freely available to any researcher wishing to use them for non-commercial purposes.
