# Supplementary material for: Novel Bacteroides Vulgatus strain protects against gluten-induced break of human celiac gut epithelial homeostasis: a pre-clinical proof-of-concept study
Source: Pediatr Res. 2024 Jan 4;95(5):1254–64. doi: 10.1038/s41390-023-02960-0 (PMC11035120; doi:10.1038/s41390-023-02960-0)

**SUPPLEMENTAL DATA**

**METHODS**

Bacterial isolation

Bacterial cell suspensions were added to an array with over 6,000 microwells (designed to act as single cell occupancy growth changes), which was then sealed with a gas permeable membrane and incubated to allow for clonal proliferation. Metabolic activity was monitored with redox indicator and fluorescence imaging. The Prospector software was then used to analyze the images, generate the transfer lists, and drive the automated, aseptic transfer of bacterial cells from array to a standard multi-well plate. A biobank of isolated bacterial strains was generated for whole genomic sequencing and functional studies.

Bacterial growth culture conditions

Several culture media were used, including Robertson's Cooked Meat (RCM) medium, Yeast Casitone Fatty Acids Broth with Carbohydrates (YCFAC BROTH), Peptone Yeast Glucose (PYG) Broth, and Brain Heart Infusion (BHI) Broth to find the ideal culture conditions for the bacteria to be cultivated. The bacterial strains of interest are all anaerobic; thus, cultures were prepared in an anaerobic chamber in order to avoid exposure to oxygen. After identifying the five strains of interest in the microwells, a small amount of bacteria was obtained from the microwell using a sterile inoculation loop and placed into a different deep microwell plate containing the different bacterial culture media. The five strains that were selected include *Bifidobacterium breve, Bacteroides thetaiotaomicron, Bifidobacterium longum, Bacteroides uniformis,* and *Bacteroides vulgatu*s. The liquid cultures were allowed to incubate at 37°C for 24 hours in an anerobic chamber. After the 24-hour incubation period, cultures were quantified by measuring the optical density at 600 nm ($OD_{600}$). The reported $OD_{600}$ readings are the following: 0.081 for *Bifidobacterium breve*, 0.6078 for *Bacteroides thetaiotaomicron*, 0.127 for *Bifidobacterium longum*, 0.6618 for *Bacteroides uniformis, and 0.8283 for Bacteroides vulgatu*s. Subcultures were then collected, centrifuged at 4000 rpm, and the supernatants were then filtered successively through a 0.4 μm and a 0.2 μm syringe filter. The cell free

supernatants (CFS) were frozen at -80.0ºC for future use while pellets were flash frozen for genomic DNA sequencing.

Bacterial sequencing and mutation analysis

*Pruning and functional categorization of mutated genes*: The list of over 20,000 mutations identified in our isolated *B. vulgatus*-A2 strain compared to the *B. vulgatus* ATCC 8482 reference strain was reviewed. Areas of missing coverage, unassigned new junctions, silent mutations, and intergenic mutations as well as additional selected genes were removed based on annotation describing their involvement in DNA or RNA processing (such as rRNAs or tRNAs). A complete list of SeqCenter annotations for these removed genes is reported in **Supplementary Figure S1**. The remaining genes with mutations were then analyzed for functional categorization. Genes with multiple mutations were counted once and the list of resulting 1,342 mutated genes was searched via UniProt online database and then categorized as bacterial barrier function, metabolism, cell replication and turnover, inflammation when interacting with host, a combination of the prior categories, or unknown.

*Automated literature search of mutated genes*: We developed an automated search tool based on the pypubmed tool (https://pypi.org/project/pypubmed/) to access the PubMed API and search if any of the 1,342 mutated genes in our isolated *B. vulgatus*-A2 strain has been reported to be implicated in interactions with host. We used this tool to iteratively search each mutated gene by using the following keywords combinations: gene + "bacteroides" + "vulgatus" + ((immune response) OR (gut barrier) OR (inflammation).

Establishing organoid-macrophages co-culture model

For this work, we utilized duodenal tissue to derive organoids at two timepoints from PCeD1 and subsequently when diagnosed with CD one year later. We also generated a second set of PCeD organoids from another patient who also had elevated serology but did not have histologic evidence of CeD (PCeD2). Healthy control organoids were from the Center for Celiac Research organoid biorepository MGHfC [52]. Organoids derived monolayers were generated as previously done [52] and monitored for confluence via light microscope and transepithelial electrical resistance (TEER, as described below) upon media changes every other day [41]. Confluence was achieved by day 8-10.

Macrophages (MΦ) derived from circulating monocytes were employed to establish the organotypic coculture, with methodological details are reported in the supplement section. The MΦ were resuspended in RPMI-1640 Medium (#R8758, Sigma Aldrich, Burlington, MA) supplemented with heat-inactivated Fetal Bovine Serum (#F4135, Sigma Aldrich, Burlington, MA) and Animal-Free Recombinant Human M-CSF (#AF-300-25, Peprotech, Cranbury, NJ), and plated at 55,000 cells per well in a 96 well tissue culture-treated high throughput screening receiver plate (#3382, Corning, Tewksbury, MA). The culture medium was changed every three to four days. After seven days in culture, the culture medium was removed and the MΦ were rinsed with DMEM/F12.

A 96 TW plate containing differentiated organoid-derived monolayers was placed on top of the receiver plate containing the MΦ, thus establishing the macrophage-epithelium organotypic model. For some comparative analyses, we had a 96 well receiver plate with only MΦ as control. After 24 hours, the permeability and cytotoxicity of the macrophage-epithelium organotypic model were measured. Gene expression analysis was performed on the MΦ from the organotypic model.

Organoid monolayer microRNAs analysis

cDNA was prepared using miRCURY® LNA® RT Kit (Cat. #:  339340, QIAGEN, Hilden, Germany) and used to perform a high throughput molecular analysis on 96-well plates of the “Human Inflammatory Response & Autoimmunity Focus” miRCURY LNA miRNA PCR Panel (cat #: YAHS-205Z, QIAGEN, Hilden, Germany). Real-time PCR reactions were run on AB 7500 System instrument (Applied Biosystems, Foster City, CA) according to manufacturer’s instructions (n = 3 for each experimental group). Following quality control test, data were normalized using NormFinder as algorithm on GeneGlobe software from QIAGEN. The relative expression of all tested miRNAs was calculated using the $2^{-\Delta\Delta Ct}$ method and was reported as fold regulation. The prediction of miRNAs targets and the gene set enrichment analysis of the predicted targets were performed for the significantly modulated miRNAs using TargetScan and Enrichr (using KEGG 2021 human database), respectively.

## RESULTS

### Gut organoid-MΦ co-culture

While most of MΦ migrate in tissues early during embryogenesis and develop locally, intestinal MΦ are constantly replenished from circulating monocytes even at steady state [53], suggesting both a different ontogeny of these cells from their resident counterparts in other tissues and the possible influence of environmental factors in promoting their differentiation. To evaluate the effect of “epithelial micromilieu” on MΦ differentiation, we evaluated multiple markers known to be differentially expressed in intestinal versus circulating monocytes including CD14, CD89, CD32 and CD64 [54]. We found that MΦ differentiated from monocytes from a healthy donor had reduced the expression of CD14 and CD64 when cultured with either organoid monolayers from HC or PCeD for 24h (**Supplementary Figure S2**) compared to MΦ alone, whereas CD32 and CD89 were below detection levels (data not shown). These data are in line with evidence supporting that the intestinal monolayer [55] contributes to the MΦ makeup toward a specific gut phenotype [54], and validate the use of the macrophage-organoid co-culture as a relevant organotypic model to study the interaction between epithelium-immune cells and environmental stimuli.

***B. vulgatus*-A2 sequencing and genomic analysis**

Examples of additional mutations in our *B. vulgatus*-A2 that may alter host-microbe interactions include: (i) a peptidoglycan hydrolase that activate host immune pathways to enhance epithelial barrier integrity (A6L2T7); (ii) the outer membrane TonB receptor family protein that polarizes host macrophages into the M2 state (A6KWM3); (iii) a conserved protein found in conjugate transposon involved in antagonistic secretion systems to aid in *Bacteroides* inter-strain competition (A6L0P3); and (iv) S-ribosylhomocysteine lyase, which helps synthesize the secreted autoinducer 2 (AI-2) used in quorum sensing, biofilm formation, and virulence factor expression (A6KYS6) [56-59]. The strain might additionally alter the intestinal microenvironment due to a mutation in an outer membrane protein involved in bile acid deconjugation (A6L4A1) or iron chelation via a ferrous iron transport protein B that associated with reduction in inflammation in ulcerative colitis patients (A6L4B2) [60-62].

**SUPPLEMENTAL TABLES AND FIGURES**

**Supplemental Table ST1:** The oligonucleotide primers used for the RT-PCR analysis

| Gene | Forward | Reverse |
|---|---|---|
| 18S | AGAAACGGCTACCACATCCA | CCCTCCAATGGATCCTCGTT |
| CCL25 | GGCCCTCATGCTGTAAAGAAG | TGCTGATGGGATTGCTAAACTT |
| CD14 | ACGCCAGAACCTTGTGAGC | GCATGGATCTCCACCTCTACTG |
| CD64 | AGCTGTGAAACAAAGTTGCTCT | GGTCTTGCTGCCCATGTAGA |
| MUC6 | CTGCCCTATACCAGCAATGGA | CTGACCCATGTACTTCCGCTC |
| CLDN18 | ACATGCTGGTGACTAACTTCTG | AAATGTGTACCTGGTCTGAACAG |

**Supplemental Figure S1**: Fold change gene expression analysis of CD14 and CD64 in MΦ co-cultured with healthy controls (1 and 2), and PCeD monolayers and compared to MΦ alone (m-CSF_MΦ). One-way ANOVA (*** )P<.001, (****)P<.0001

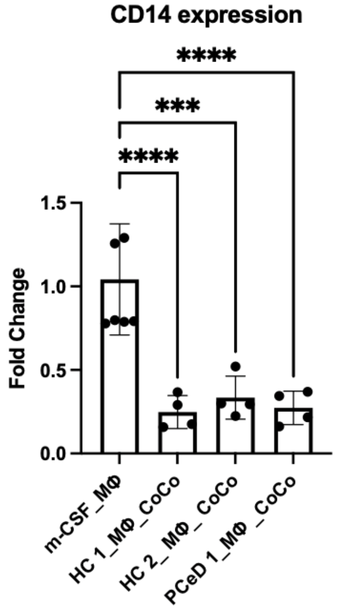


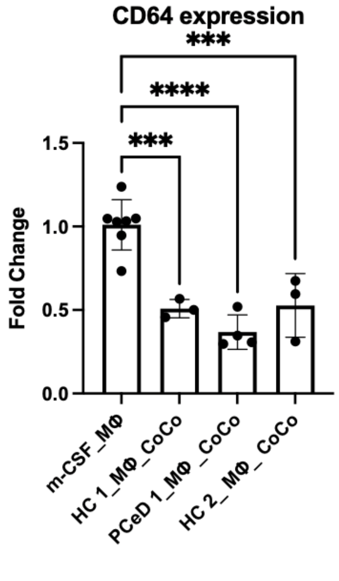

**Supplemental Figure S2:** Paracellular permeability evaluated by passage of FITC-PEG550 across monolayers in PCeD coculture upon 10% CFS treatment before being exposed to PTG[SDS4] for four hours. One-way ANOVA.

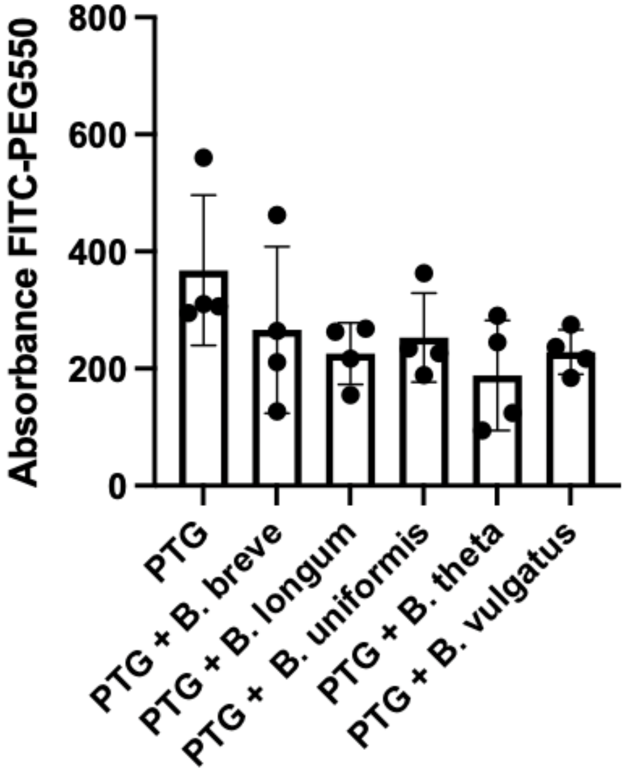

**Supplemental Figure S3: List of additional genetic mutations is *B. vulgatus*-A2 compared to reference strain.** In addition to areas labeled unassigned new junctions, silent mutations, and intergenic mutations selected genes related to 16S and 23S ribosomal RNA, DNA primases, DNA polymerases, RNA polymerases, exonucleases, translocation elements, topoisomerases, helicases, transposases, RNA sigma factors, and tRNAs were from the original variant analysis list, thus generating the list of 1,342 unique genes with mutations mentioned in the body of the article that were manually and computationally analyzed.

16S ribosomal RNA/2TM domain-containing protein
16S ribosomal RNA/ABC transporter permease
16S ribosomal RNA/hypothetical protein
16S rRNA (adenine(1518)-N(6)/adenine(1519)-N(6))- dimethyltransferase RsmA
16S rRNA (cytosine(1402)-N(4))-methyltransferase RsmH
23S ribosomal RNA
23S ribosomal RNA/5S ribosomal RNA
23S rRNA (adenine(2503)-C(2))-methyltransferase RlmN
23S rRNA (guanosine(2251)-2'-O)-methyltransferase RlmB
23S rRNA (uracil(1939)-C(5))-methyltransferase RlmD
30S ribosomal protein S1/SIMPL domain-containing protein
30S ribosomal protein S10/elongation factor G
30S ribosomal protein S15/hypothetical protein
3'-5' exonuclease domain-containing protein 2
3'-5' exonuclease/DNA polymerase III subunit beta
50S ribosomal protein L11 methyltransferase
50S ribosomal protein L2
50S ribosomal protein L3/30S ribosomal protein S10
50S ribosomal protein L34/anaerobic sulfatase-maturation protein
5'-methylthioadenosine/adenosylhomocysteine nucleosidase
5'-methylthioadenosine/adenosylhomocysteine nucleosidase
5S ribosomal RNA/2-isopropylmalate synthase
5S ribosomal RNA/2-isopropylmalate synthase
cysteine--tRNA ligase
cysteine--tRNA ligase/aromatic amino acid ammonia-lyase
Cys-tRNA(Pro) deacylase
D-aminoacyl-tRNA deacylase/excinuclease ABC subunit UvrC
D-aminoacyl-tRNA deacylase/excinuclease ABC subunit UvrC
DNA alkylation repair protein
DNA gyrase subunit A
DNA gyrase subunit A/ATP-dependent Clp protease ATP-binding subunit
DNA gyrase subunit A/ATP-dependent Clp protease ATP-binding subunit
DNA gyrase subunit A/ATP-dependent Clp protease ATP-binding subunit
DNA helicase RecQ/helix-turn-helix domain-containing protein
DNA helicase RecQ/helix-turn-helix domain-containing protein
DNA polymerase I
DNA polymerase III subunit gamma/tau
DNA polymerase IV
DNA polymerase IV/tRNA-Pro
DNA primase
DNA primase
DNA repair protein RadA/bifunctional aspartate kinase/homoserine dehydrogenase I

DNA repair protein RadC
DNA repair protein RecN/bifunctional phosphopantothenoylcysteine decarboxylase/phosphopantothenate--cysteine ligase CoaBC
DNA repair protein RecO
DNA repair protein RecO/GatB/YqeY domain-containing protein
DNA topoisomerase (ATP-hydrolyzing) subunit B/30S ribosomal protein S20
DNA topoisomerase 3
DNA/RNA non-specific endonuclease/alpha-N-arabinofuranosidase
DNA-directed RNA polymerase subunit alpha
DNA-directed RNA polymerase subunit omega/DUF4293 domain-containing protein
DNA-processing protein DprA
EFR1 family ferrodoxin/50S ribosomal protein L20
endonuclease
endonuclease MutS2
endonuclease/exonuclease/phosphatase family protein
excinuclease ABC subunit UvrA
excinuclease ABC subunit UvrB
excinuclease ABC subunit UvrC/adenine phosphoribosyltransferase
exodeoxyribonuclease III/hypothetical protein
exodeoxyribonuclease VII small subunit
exonuclease SbcCD subunit D C-terminal domain-containing protein
helicase
helicase/DsbA family protein
helicase/hypothetical protein
helicase-related protein/hypothetical protein
helix-turn-helix domain-containing protein/isoleucine--tRNA ligase
helix-turn-helix transcriptional regulator/DNA polymerase III subunit delta
hypothetical protein
hypothetical protein/50S ribosomal protein L17
hypothetical protein/50S ribosomal protein L9
hypothetical protein/5S ribosomal RNA
IS1182 family transposase
IS1182 family transposase/GNAT family N-acetyltransferase
IS1182 family transposase/hypothetical protein
IS1182 family transposase/TonB-dependent receptor
IS1595-like element ISBvu2 family transposase
IS1595-like element ISBvu2 family transposase
IS1595-like element ISBvu2 family transposase/hypothetical protein
IS21 family transposase
IS21 family transposase
IS21 family transposase/hypothetical protein
IS256 family transposase
IS66 family insertion sequence element accessory protein TnpB
IS66 family insertion sequence element accessory protein TnpB/acyltransferase
IS66 family insertion sequence element accessory protein TnpB/RagB/SusD family nutrient uptake outer membrane protein
IS66 family transposase/hypothetical protein
IS982 family transposase/B12-binding domain-containing radical SAM protein
IS982 family transposase/IS21 family transposase
IS982 family transposase/phosphoglucosamine mutase
ISAs1 family transposase

ISAs1 family transposase/ATP-binding protein
isoleucine--tRNA ligase
leucine--tRNA ligase
lysine--tRNA ligase
methionine--tRNA ligase
NAAT family transporter/5S ribosomal RNA
nucleoside-diphosphate kinase/ATP-dependent DNA helicase RecG
nucleotide exchange factor GrpE
phenylalanine--tRNA ligase subunit beta
phosphoribosylaminoimidazolecarboxamide formyltransferase/16S ribosomal RNA
replicative DNA helicase/phenylalanine--tRNA ligase subunit beta
restriction endonuclease
restriction endonuclease subunit M
restriction endonuclease subunit S
ribonuclease Z
ribonucleoside-diphosphate reductase subunit alpha
ribonucleoside-diphosphate reductase subunit alpha/hypothetical protein
ribonucleotide-diphosphate reductase subunit beta
ribonucleotide-diphosphate reductase subunit beta/ribonucleoside-diphosphate reductase subunit alpha
ribosome maturation factor RimM
RNA degradosome polyphosphate kinase
RNA methyltransferase
RNA methyltransferase/alcohol dehydrogenase
RNA methyltransferase/outer membrane beta-barrel protein
RNA polymerase factor sigma-54/Xaa-Pro aminopeptidase
RNA polymerase sigma factor
RNA polymerase sigma factor
RNA polymerase sigma-70 factor
RNA polymerase sigma factor RpoD/SigA/hypothetical protein
RNA polymerase sigma-70 factor
RNA polymerase sigma-70 factor/alpha-galactosidase
RNA polymerase sigma-70 factor/FecR domain-containing protein
RNA polymerase sigma-70 factor/GH92 family glycosyl hydrolase
RNA polymerase sigma-70 factor/ISAs1 family transposase
RNA polymerase sigma-70 factor/outer membrane beta-barrel protein
RNA polymerase sigma-70 factor/SusC/RagA family TonB-linked outer membrane protein
RNase P RNA component class A
RNase P RNA component class A/chaperone modulator CbpM
Rne/Rng family ribonuclease/N-acetylmuramoyl-L-alanine amidase
Rpn family recombination-promoting nuclease/putative transposase
Rpn family recombination-promoting nuclease/putative transposase/30S ribosomal protein S16
rRNA cytosine-C5-methyltransferase
rRNA maturation RNase YbeY
S-adenosylmethionine:tRNA ribosyltransferase-isomerase
sel1 repeat family protein/sigma-54 dependent transcriptional regulator
sigma-54 dependent transcriptional regulator
sigma-54 dependent transcriptional regulator/glycine betaine/L-proline ABC transporter ATP-binding protein
sigma-70 family RNA polymerase sigma factor
sigma-70 family RNA polymerase sigma factor/DUF4974 domain-containing protein

sigma-70 family RNA polymerase sigma factor/glycoside hydrolase 43 family protein
sigma-70 family RNA polymerase sigma factor/NigD-like protein
sigma-70 family RNA polymerase sigma factor/ROK family transcriptional regulator
single-stranded DNA-binding protein
site-specific integrase
site-specific integrase/AraC family transcriptional regulator
site-specific integrase/Holliday junction branch migration DNA helicase RuvB
site-specific integrase/hypothetical protein
site-specific integrase/site-specific integrase
site-specific integrase/TonB-dependent receptor
site-specific integrase/transcriptional regulator
threonine--tRNA ligase
TIGR03915 family putative DNA repair protein
transcription-repair coupling factor
transcription-repair coupling factor/TonB family protein
translocation/assembly module TamB
translocation/assembly module TamB domain-containing protein
transposase
transposase/hypothetical protein
transposase/IS21 family transposase
transposase/IS66 family insertion sequence element accessory protein TnpB
transposase/tRNA-Leu
tRNA (5-methylaminomethyl-2-thiouridine)(34)-methyltransferase MnmD
tRNA (5-methylaminomethyl-2-thiouridine)(34)-methyltransferase MnmD/hypothetical protein
tRNA (adenosine(37)-N6)-dimethylallyltransferase MiaA
tRNA (adenosine(37)-N6)-threonylcarbamoyltransferase complex ATPase subunit type 1 TsaE
tRNA (guanosine(37)-N1)-methyltransferase TrmD
tRNA (guanosine(37)-N1)-methyltransferase TrmD/dihydroorotate dehydrogenase
tRNA (N(6)-L-threonylcarbamoyladenosine(37)-C(2))- methylthiotransferase MtaB
tRNA (N(6)-L-threonylcarbamoyladenosine(37)-C(2))- methylthiotransferase MtaB/long-chain fatty acid--CoA ligase
tRNA 2-thiocytidine biosynthesis TtcA family protein/PD40 domain-containing protein
tRNA 2-thiouridine(34) synthase MnmA
tRNA dihydrouridine synthase DusB
tRNA dihydrouridine synthase DusB/DUF2807 domain-containing protein
tRNA lysidine(34) synthetase TilS
tRNA lysidine(34) synthetase TilS/tetratricopeptide repeat protein
tRNA pseudouridine(38-40) synthase TruA
tRNA uridine-5-carboxymethylaminomethyl(34) synthesis enzyme MnmG
tRNA uridine-5-carboxymethylaminomethyl(34) synthesis GTPase MnmE
tRNA uridine-5-carboxymethylaminomethyl(34) synthesis GTPase MnmE/hypothetical protein
tRNA-Arg/4-hydroxy-tetrahydrodipicolinate synthase
tRNA-Arg/left-handed beta-roll domain-containing protein
tRNA-Asn/hybrid sensor histidine kinase/response regulator transcription factor
tRNA-Asn/tRNA-Asn
tRNA-Asp/tRNA-Asp
tRNA-Cys/ferrous iron transport protein B
tRNA-dihydrouridine synthase family protein
tRNA-dihydrouridine synthase family protein/DUF3874 domain-containing protein
tRNA-Gln/DUF349 domain-containing protein
tRNA-Gln/flavin reductase

tRNA-Glu/DcaP family trimeric outer membrane transporter
tRNA-Glu/DNA repair protein RecO
tRNA-Glu/tRNA-Ser
tRNA-Gly/hypothetical protein
tRNA-Leu
tRNA-Leu/DUF5110 domain-containing protein
tRNA-Leu/tRNA-Gly
tRNA-Leu/tRNA-Leu
tRNA-Lys
tRNA-Lys
tRNA-Lys/AI-2E family transporter
tRNA-Lys/magnesium/cobalt transporter CorA
tRNA-Met/tRNA-Met
tRNA-Pro/tRNA-Phe
tRNA-Ser/DNA polymerase III subunit gamma/tau
tRNA-Ser/glycosyltransferase
tRNA-Ser/hypothetical protein
tRNA-Thr/DEAD/DEAH box helicase
type I DNA topoisomerase
type I restriction endonuclease subunit R
type I restriction enzyme HsdR N-terminal domain-containing protein/C4-type zinc ribbon domain-containing protein
type I restriction-modification system subunit M
tyrosine-type recombinase/integrase
type II pantothenate kinase/5S ribosomal RNA
U32 family peptidase
uracil-DNA glycosylase
uracil-DNA glycosylase family protein
UvrD-helicase domain-containing protein
valine--tRNA ligase
YccF domain-containing protein/50S ribosomal protein L11 methyltransferase
response regulator/IS66 family transposase
CCA tRNA nucleotidyltransferase

**Supplemental Figure S4:** Functional categorization of the 1,342 mutated genes in our isolated *B. vulgatus*-A2 according to UniProt annotations.

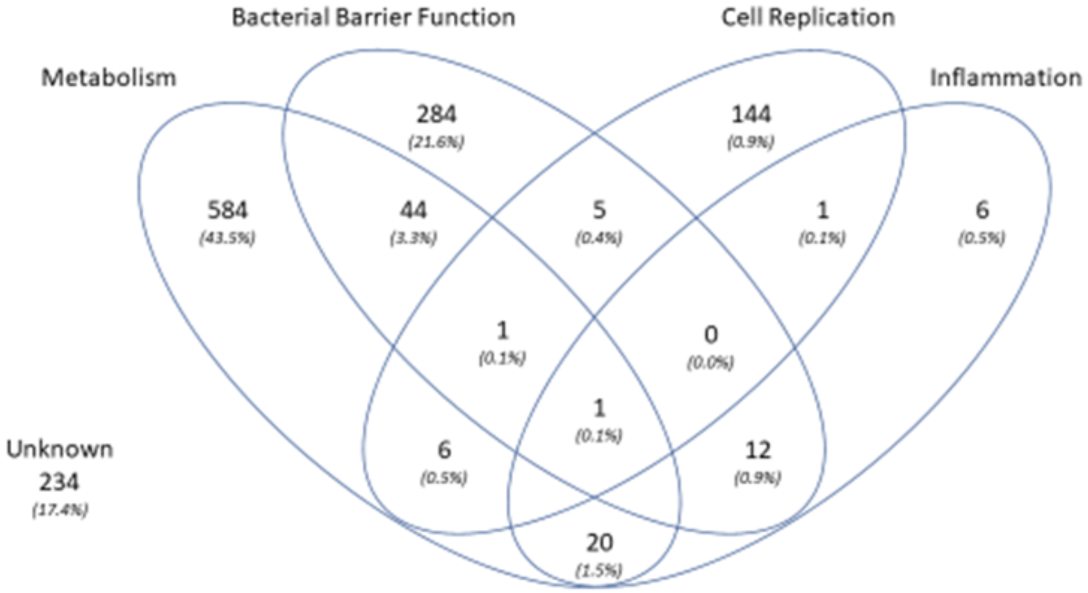

**Supplemental Figure S5.** Effect of *B. vulgatus*-A2 CFS on let-7c-5p expression in HC organoids. High-throughput miRNAs analysis was performed by Real-Time PCR in *B. vulgatus*-A2 CFS-treated HC organoids (white bars) vs. untreated HC organoids (black bars) using pre-custom plates for the inflammatory response and the autoimmunity focus (n = 3 organoids derived monolayers/group). Histograms represent the mean ± SEM. ****P< 0.0001.

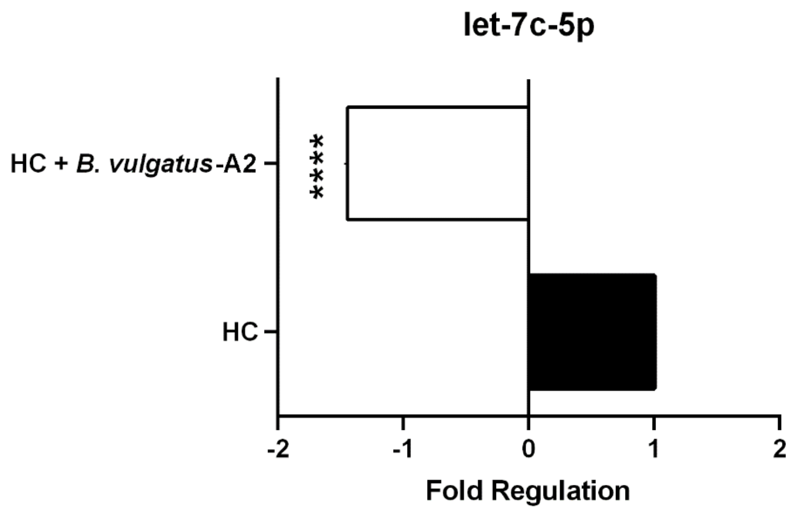

**Supplemental Figure S6**. Gene set enrichment analysis of the predicted miRNA targets was performed by Enrichr using KEGG 2021 human database - bars were sorted by the p-value ranking. The preliminary target prediction was done by TargetScan for the miRNAs resulted to be significantly modulated by a high-throughput Real-Time PCR analysis using pre-custom plates for the inflammatory response and the autoimmunity focus in PCeD and HC organoids with and without *B. vulgatus*-A2 CFS treatment.

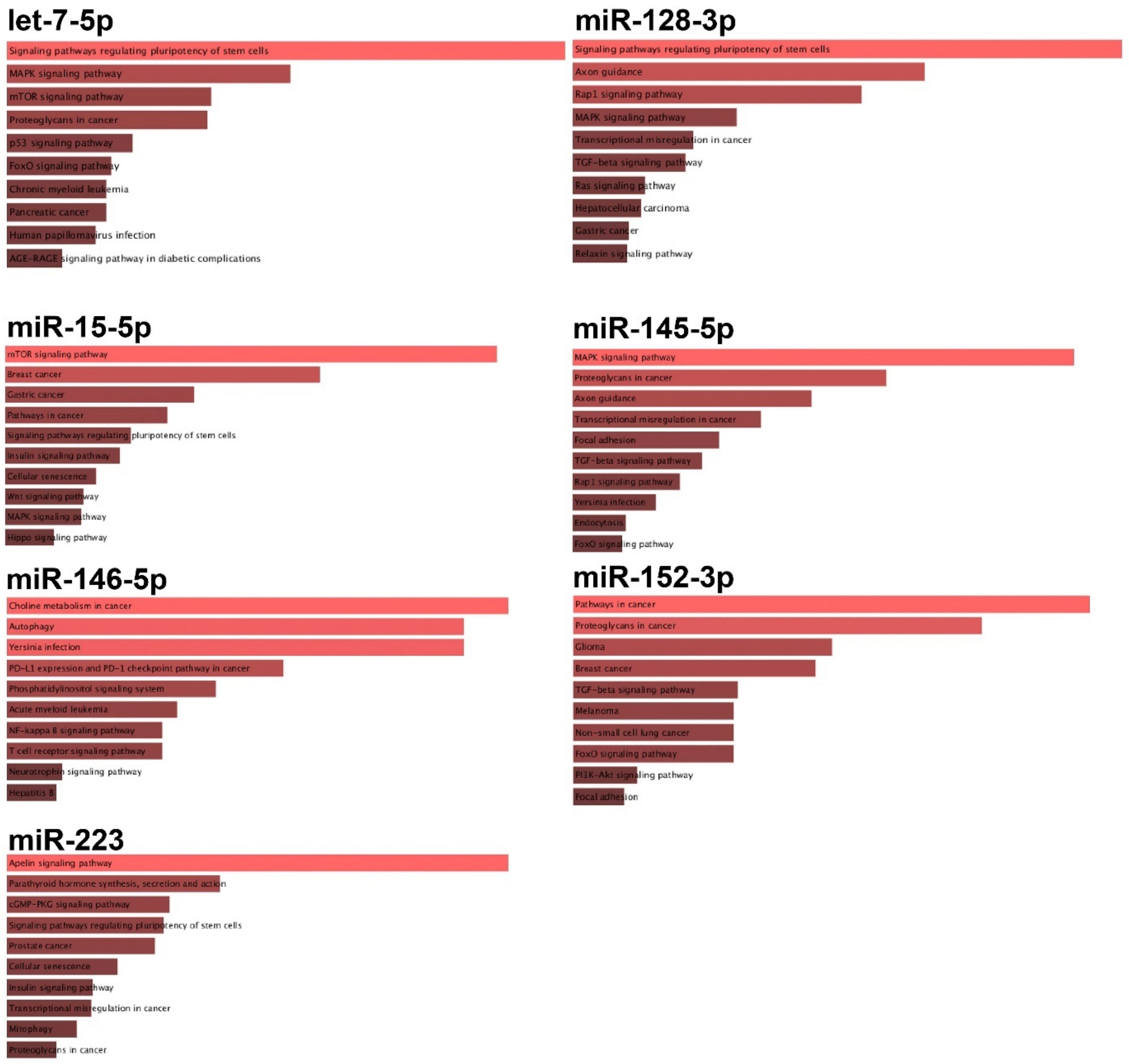

Supplement: Supplementary file 1 — Supplemental Data [file 41390_2023_2960_MOESM1_ESM.pdf]
